# Uncovering Ecosystem Service Bundles through Social Preferences

**DOI:** 10.1371/journal.pone.0038970

**Published:** 2012-06-18

**Authors:** Berta Martín-López, Irene Iniesta-Arandia, Marina García-Llorente, Ignacio Palomo, Izaskun Casado-Arzuaga, David García Del Amo, Erik Gómez-Baggethun, Elisa Oteros-Rozas, Igone Palacios-Agundez, Bárbara Willaarts, José A. González, Fernando Santos-Martín, Miren Onaindia, Cesar López-Santiago, Carlos Montes

**Affiliations:** 1 Social-Ecological Systems Laboratory, Department of Ecology, Universidad Autónoma de Madrid, Madrid, Spain; 2 Department of Plant Biology and Ecology, Universidad de Almería, Almería, Spain; 3 Plant Biology and Ecology Department – Universidad del País Vasco UPV/EHU, Bizkaia, Spain; 4 Institute of Environmental Science and Technology, Faculty of Sciences, Universitat Autònoma de Barcelona, Bellaterra–Cerdanyola del Vallés, Spain; 5 Research Center for the Management of Agricultural and Environmental Risks (CEIGRAM), Universidad Politécnica de Madrid, Madrid, Spain; University of Massachusetts, United States of America

## Abstract

Ecosystem service assessments have increasingly been used to support environmental management policies, mainly based on biophysical and economic indicators. However, few studies have coped with the social-cultural dimension of ecosystem services, despite being considered a research priority. We examined how ecosystem service bundles and trade-offs emerge from diverging social preferences toward ecosystem services delivered by various types of ecosystems in Spain. We conducted 3,379 direct face-to-face questionnaires in eight different case study sites from 2007 to 2011. Overall, 90.5% of the sampled population recognized the ecosystem’s capacity to deliver services. Formal studies, environmental behavior, and gender variables influenced the probability of people recognizing the ecosystem’s capacity to provide services. The ecosystem services most frequently perceived by people were regulating services; of those, air purification held the greatest importance. However, statistical analysis showed that socio-cultural factors and the conservation management strategy of ecosystems (i.e., National Park, Natural Park, or a non-protected area) have an effect on social preferences toward ecosystem services. Ecosystem service trade-offs and bundles were identified by analyzing social preferences through multivariate analysis (redundancy analysis and hierarchical cluster analysis). We found a clear trade-off among provisioning services (and recreational hunting) versus regulating services and almost all cultural services. We identified three ecosystem service bundles associated with the conservation management strategy and the rural-urban gradient. We conclude that socio-cultural preferences toward ecosystem services can serve as a tool to identify relevant services for people, the factors underlying these social preferences, and emerging ecosystem service bundles and trade-offs.

## Introduction

The ecosystem services concept has been increasingly used by academics, researchers and policy-makers [1], [2] to support and inform environmental management and biodiversity conservation strategies [3], [4]. Most studies have focused either on biophysical assessments of the capacity of ecosystems to deliver services (e.g., [5]–[8]), or on the economic value of ecosystem services (e.g., [8]–[11]). Few studies, however, have addressed socio-cultural preferences toward ecosystem services from the perspective of human values, attitudes, and beliefs while using a non-economic approach [12]. A non-economic evaluation offers ways of understanding the motivations underlying social preferences toward ecosystem services, thereby unraveling values that tend to be obscured by monetary languages [13], [14].

Because ecosystem service assessments are determined by analyzing the effect of ecosystems and biodiversity on human well-being, it is necessary to understand the ways society benefits from nature and, hence, the many reasons that societies value ecosystem services [15], [16]. Identifying the reasons and motivations for protecting ecosystem services helps to understand which services are relevant for different stakeholders and which trade-offs need to be addressed when making decisions regarding land-use management [17]. Trade-offs can arise from the different interests of social agents involved because one ecosystem may be valued differently by different stakeholders in relation to its capacity to provide services that fulfill their own interests. For instance, a wetland is likely to be valued by fishermen primarily for its capacity to maintain the abundance of specific game fish species, by farmers for its ability to supply water for irrigation, by conservationists for their capacity to provide habitat for endangered and rare wildlife species, and by nature tourists for its capacity to provide recreation and aesthetic enjoyment [8], [18].

Based on socio-cultural preferences, the concept of ecosystem service bundles emerge as a useful tool for identifying ecosystem service synergies and trade-offs [19], [20] resulting from stakeholders’ diverging interests and knowledge. Given the growing demand for the incorporation of the socio-cultural dimension of ecosystem services in environmental policy agendas [15], [21], [22], understanding social preferences toward the protection of ecosystem services has become a research priority [16]. To our knowledge, no empirical studies have addressed ecosystem service bundles based on socio-cultural preferences, and few studies have analyzed the stakeholders’ preferences toward several services (see Table 1). Therefore, there is a specific need to explore social preferences and perceptions toward ecosystem services in the context of current scientific and environmental policy interests at international organization levels (i.e., Millennium Ecosystem Assessment follow-up; The Economics of Ecosystems and Biodiversity (TEEB) [10]; Intergovernmental Science-Policy Platform on Biodiversity and Ecosystem Services (IPBES) [23]; or the Convention of Biological Diversity’s (CBD) 2020 targets) as well as national organization levels (i.e., the Millennium Ecosystem Assessment of Spain [24] and the Spanish law 42/2007, on Natural Heritage and Biodiversity).

**Table 1:** Studies analyzing social perceptions of ecosystem services.

Source	Type of ecosystems(after [71])	Category of ecosystem services (after [71])	Study area	Methodology	Stakeholderssampled
Martín-López, 2007 [72]	Wetlands	Provisioning; Regulating; Cultural	Doñana Protected Area,Spain	Face-to-face questionnaires	Local people, visitors, environmental experts
Rönnbäck, 2007 [49]	Coastal system (mangroves)	Provisioning; Regulating; Cultural	Gazi and Makongeni, Kenia	Semi-structured interviews	Local people
Agbenyega, 2008 [30]	Forests	Supporting; Provisioning; Regulating; Cultural	Eastern England, UK	Questionnaires	Local people
Iftekhar, 2008 [32]	Wetlands	Supporting; Provisioning; Regulating; Cultural	Nijhum Dwip, Bangladesh	Individual interviews andgroup meetings	Local people andkey informants
Sodhi, 2009 [33]	Forests	Provisioning; Regulating; Cultural	Forested parks in Myanmar, Philippines, and Thailand	Individual interviews	Local people
Hartter, 2010 [31]	Forests andwetlands	Provisioning; Regulating	The Kibale National Park, Uganda	Semi-structured interviews	Local people
Zheng, 2010 [34]	Drylands	Provisioning; Regulating; Cultural	Mongolia Plateau	Individual interviews and face-to-face questionnaires	Local people
Castro, 2011 [73]	Drylands	Provisioning; Regulating; Cultural	Almería, Spain	Face-to-face questionnaires	Local people, visitors, environmental experts
Lamarque, 2011 [26]	Grasslands ofmountains	Provisioning; Regulating; Cultural	French Alps, Austrian Alps,and English uplands	Individual and groupinterviews	Regional experts andlocal farmers
Vilardy, 2011 [74]	Coastal wetland	Provisioning; Regulating; Cultural	Ciénaga Grande of SantaMarta, Colombia	Semi-structured interviewsand expert meetings	Local people and environmental experts
Warren-Rhodes,2011 [50]	Coastal system (mangroves)	Provisioning; Regulating; Cultural	Solomon Islands	Semi-structured interviews	Local people
Calvet-Mir, 2012 [27]	Home gardens	Provisioning; Regulating; Cultural	Catalan Pyrenees, Spain	Semi-structured interviewsand questionnaires	Local people, visitors,and scientists

In this study, we analyzed socio-cultural preferences toward ecosystem services delivered by different types of Spanish ecosystems and how they can promote ecosystem service trade-offs and bundles. Here, the term “socio-cultural preferences” incorporates individual perceptions, knowledge, and associated values [25]. In doing so, we specifically explore the following: (*i*) the probability that people recognize the capacity of ecosystems to deliver services to society and the factors influencing such recognition; (*ii*) the relative importance given by people to different categories of ecosystem services (i.e., provisioning, regulating, and cultural) and their underlying factors; (*iii*) the factors that affect the relative importance stakeholders give to particular ecosystem service and the potential trade-offs emerging; and (*iv*) the ecosystem service bundles that can emerge from diverging socio-cultural preferences.

## Materials and Methods

### Study Sites

**Figure 1:**
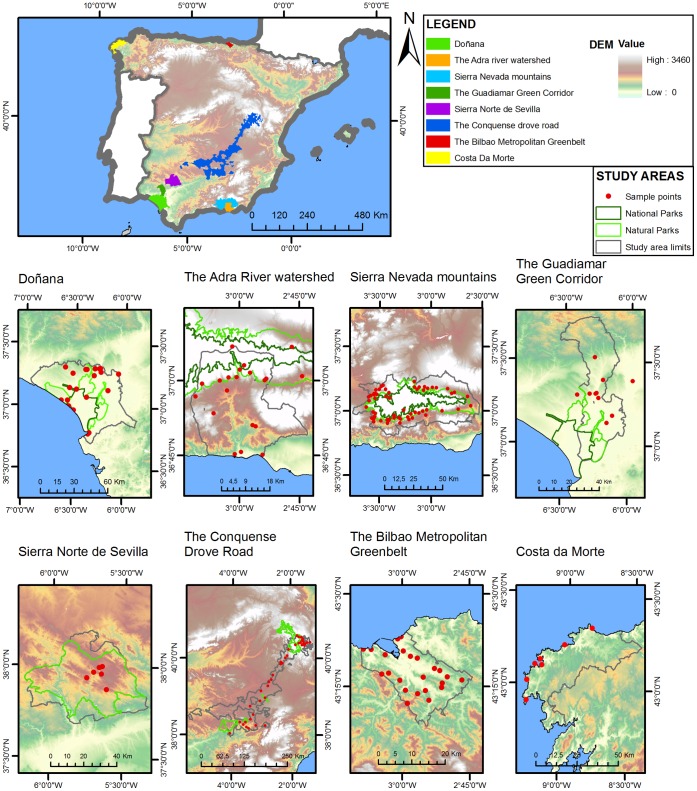
Study areas. Sample points are indicated with red circles. National and Natural Parks are shown in dark and light green, respectively.

The research was conducted at eight sites in the Iberian Peninsula (Figure 1) to capture (*i*) a representative sample of ecosystem diversity and (*ii*) different environmental management strategies (i.e., National Parks with a strict conservation level, Natural Parks with a medium conservation level that allows traditional and cultural management practices, and non-protected areas). Thus, both biophysical and socio-cultural variability were considered to select case sites because they determine a different supply and demand of ecosystem services [26]. For more details regarding case study sites, see Supplementary Information (Figure S1 and Table S1).

### Data Sampling

Data sampling was conducted in each of the eight case sites by following a consistent survey design. The population sampled was randomly selected to cover a wide range of ecosystem service beneficiaries, such as local inhabitants, visitors, or environmental technical experts. The sampling population was restricted to individuals over 18 years old. A total of 3379 direct face-to-face questionnaires were conducted between 2007 and 2011. To test the suitability of the questionnaire design, we conducted a preliminary sampling in each case study. More details about the sampling characteristics are provided in Table S1, and further details of the sampling population are provided in Table S2.

The questionnaire was structured in four sections, regarding the following topics: (*i*) the respondents’ previous knowledge about the studied ecosystem’s capacity to provide services to society; (*ii*) the respondents’ perception of the importance of ecosystem services for the well-being of people in the area, after pollsters provided information about the services potentially delivered by ecosystems; (*iii*) the individuals’ environmental behavior (i.e., if the respondent was a member of either an environmental or social association and if the respondent had visited any protected areas during the previous year); and (*iv*) socio-demographic characteristics of the surveyed individuals (i.e., place of residence, formal education, age, gender, and monthly income). While conducting the surveys, the term ecosystem service was always referred to as “the benefits that the ecosystems of the area provide for human well-being” to make the term more understandable and to avoid educational biases. In addition, to facilitate interpretation, a list of ecosystem services with related pictures was presented to respondents in the second portion (*ii*) of the questionnaire [27].

The variables obtained from the questionnaire are shown in the supplementary material (Table S3).

### Statistical Analysis

We used binomial logit regression to predict the probability that a respondent recognized an ecosystem’s capacity to deliver services to society. The dependent variable was coded as ‘1’ if the respondent recognized an ecosystem’s capacity to supply services and as ‘0’ if the respondent did not. Akaike’s Information Criteria (AIC) was used to select the best model among all the possible combinations of independent variables [28]. To validate the prediction of the regression, we used 250 randomly selected observations. Then, we calculated the percentage of observations where the model correctly predicted the 0–1 responses (% well classified). We also tested whether the probability of a respondent to acknowledge an ecosystem’s capacity to provide services to society differed among ecosystem types, using chi-squared contingency tests.

We used the chi-squared test to analyze the relative importance of different service categories for respondents’ well-being, comparing the number of ecosystem services identified by the respondents in each of the service categories (i.e., provisioning, regulating, and cultural). Then, we used the non-parametric Kruskal-Wallis test followed by Dunn’s multiple comparison test to analyze whether the relative importance given to each service category by respondents was affected by social (i.e., formal education) and management factors (i.e., if the ecosystem under analysis was protected by a National Park, by a Natural Park, or was a non-protected area). We also performed a non-parametric Mann-Whitney *U*-test to analyze whether the relative importance of different service categories differed with gender, rural versus urban population, elderly versus younger people, and between respondents showing environmental behavior versus those who did not.

A redundancy analysis (RDA) was used to identify socio-cultural factors associated with the relative importance of particular ecosystem services by relating ecosystem services to socio-demographic and environmental behavior variables, as well as the management strategy and ecosystem type. A Monte Carlo permutation test (1000 permutations) was performed to determine the significance of independent variables in determining the relative importance of ecosystem services. The factors with the highest inertia were used to identify ecosystem service bundles using a hierarchical cluster analysis. We used Ward’s linkage method with Euclidean distances to identify relatedness among ecosystem service preferences [29].

## Results

### Probability of Recognizing the Ecosystem’s Capacity to Deliver Services to Society

Overall, 90.5% of the sampled population recognized that ecosystems can deliver services to society. According to the logit model, factors affecting the probability that respondents recognized the ecosystem’s capacity to deliver services were the level of formal education, environmental behavior, and gender (Table 2). Our results indicate that respondents were more likely to recognize the ecosystem’s capacity to supply services when they have a higher level of formal education, higher environmental behavior, and if they were female. From the validation dataset (*N* = 250), we obtained that 92.8% of the answers were correctly classified.

**Table 2:** Logit regression for respondents’ recognition of an ecosystem’s capacity to provide services.

Variables	Coefficient	Standard deviation	*z*	*p*> |*z*|	[95% C.I.]	
Constant	1.156	0.999	1.362	0.234	−0.785	3.096
Education	0.385	0.066	33.557	<0.0001	0.254	0.515
Female	0.212	0.130	2.669	0.072	−0.042	0.466
Organization	0.572	0.247	5.335	0.021	0.087	1.057
PAs	0.423	0.128	10.856	0.001	0.171	0.674
*N* = 3129						
Log-likelihood = 1892.67, Wald Chi-squared = 72.23, (*p*> Chi^2^) <0.0001						
AIC = 1904.67						
Percentage of correct estimated predictions (%) = 90.47%						

C.I. refers to its 95% confidence.

PAs  =  If respondent visited protected areas during the previous year.

**Figure 2:**
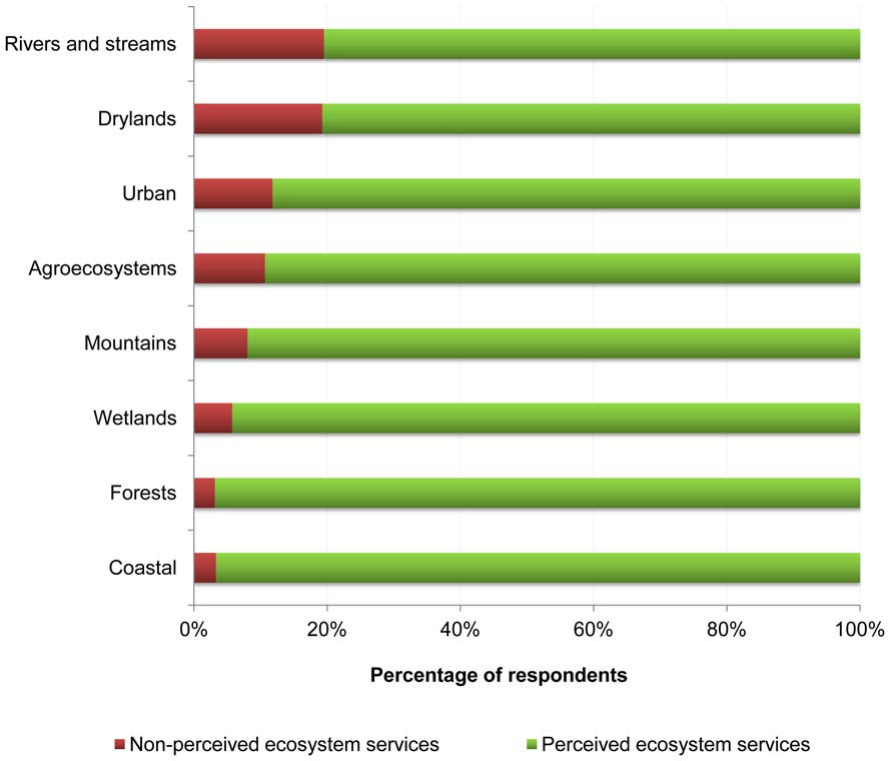
Perception of stakeholders regarding an ecosystem’s capacity to provide services. Ecosystem classification based on the Millennium Ecosystem Assessment [71].

We also found differences in the stakeholders’ recognition regarding the capacity to provide services among different types of ecosystems (*χ* ^2^ = 79.8, d.f. = 7, *p*<0.0001). While the scores regarding the recognition of an ecosystem’s capacity to supply services were high for coastal systems, forests, and wetlands, these scores were significantly lower for various ecosystems, including rivers and streams, drylands, and urban systems (Figure 2).

### Factors Underlying the Relative Importance given to Categories of Ecosystem Services

We found significant differences in the social perception about the relative importance of different ecosystem service categories (*χ* ^2^ = 885.4, d.f. = 2, *p*<0.0001): regulating services showed the highest saliency (44% of total respondents), followed by cultural services (33%), and provisioning services (23%). When respondents were asked to identify the relative importance of particular services, more than 40% identified air purification, the existence value of biodiversity, and nature tourism, as the most important services (*χ* ^2^ = 3522.2, d.f. = 13, *p*<0.0001). Few respondents seemed to perceive the role of ecosystems as providers of forests products (13% of respondents) or hunting as a recreational activity (11%). Table 3 provides a summary of descriptive statistics of the particular ecosystem services perceived by the respondents.

**Table 3:** Descriptive statistics of respondents’ preferences toward ecosystem services.

Ecosystem services	*N*	Mean (%)	S.D.
***Provisioning services***			
Agriculture	905	26.8	0.44
Cattle	788	23.3	0.42
Fishing	724	21.4	0.42
Forest products	430	12.7	0.33
***Regulating services***			
Micro-climate regulation	1071	31.7	0.46
Air purification	1522	45.0	0.49
Water regulation	1297	38.4	0.48
Soil formation	938	27.8	0.45
***Cultural services***			
Nature tourism	1392	41.2	0.49
Aesthetic values	605	17.9	0.38
Environmental education	906	26.8	0.44
Local ecological knowledge	913	27.0	0.44
Recreational hunting	358	10.6	0.30
Existence value	1420	42.0	0.49

S.D. refers to standard deviation.

The importance of different ecosystem service categories varied significantly among respondents depending on their level of formal education, gender, place of residence (i.e., urban vs. rural), age, and reported level of environmental behavior (Table 4). While rural and elderly people (i.e., more than 70 years old) mostly acknowledged provisioning services, urban and younger people (i.e., less than 30 years old) mostly acknowledged regulating services. Overall, males mostly perceived provisioning services and females mostly perceived regulating services. Additionally, people with a lower level of formal education placed more value on provisioning services (Table 4).

**Table 4:** Factors influencing people’s awareness of different ecosystem service categories.

Factors		Mean relative value (S.D.)		
		*Provisioning*	*Regulating*	*Cultural*
***Environmental behavior***				
PAs	Visitor	0.184 (0.20)	0.356 (0.27)	0.214 (0.18)
	Non-visitor	0.208 (0.21)	0.318 (0.27)	0.261 (0.18)
	*U*	1 245 269.0^***^	1 294 822.0^***^	1 358 297.0^***^
Organization	Membership	0.197 (0.19)	0.281 (0.26)	0.211 (0.18)
	Non-membership	0.177 (0.19)	0.344 (0.27)	0.221 (0.18)
	*U*	590 526.0^*^	647 158.5^***^	528 289.5
***Socio-economic***				
Place of residence	Rural	0.240 (0.22)	0.300 (0.27)	0.299 (0.18)
	Urban	0.183 (0.21)	0.428 (0.27)	0.300 (0.18)
	*U*	1 623 990.5^***^	996 411.0^***^	1 375 845.0
Level of education	None	0.293^c^ (0.24)	0.370 (0.27)	0.228^a^ (0.18)
	Primary	0.240^b,c^ (0.22)	0.350 (0.27)	0.253^b^ (0.18)
	Secondary	0.215^a,b^(0.21)	0.365 (0.28)	0.254^b^ (0.18)
	University	0.194^a^ (0.21)	0.351 (0.27)	0.250^b^ (0.19)
	*χ* ^2^	40.53^***^	3.21	19.77^***^
Age	>30 years	0.221 (0.21)	0.347 (0.28)	0.242 (0.18)
	<30 years	0.203 (0.21)	0.377 (0.28)	0.244 (0.18)
	*U*	1 265 767.5^***^	1 137 165.0^***^	1 094 182.0
	>70 years	0.273 (0.24)	0.317 (0.24)	0.250 (0.18)
	<70 years	0.213 (0.22)	0.358 (0.28)	0.242 (0.18)
	*U*	203 202.0^**^	249 135.5	224 874.5
Gender	Male	0.217 (0.22)	0.348 (0.28)	0.241 (0.185)
	Female	0.210 (0.22)	0.368 (0.27)	0.245 (0.185)
	*U*	1 390 008.5^***^	1 318 087.0^***^	1 363 616.0
***Management strategy***				
	National Park	0.196^a^ (0.19)	0.333^b^ (0.26)	0.198^a^ (0.17)
	Natural Park	0.230^a^ (0.24)	0.430^c^ (0.31)	0.277^c^ (0.17)
	Non-protected	0.223^a^ (0.21)	0.320^a^ (0.25)	0.254^b^ (0.19)
	*χ* ^2^	6.34^*^	74.97^***^	150.85^***^

S.D.  =  standard deviation.

PAs  =  If respondent visited protected areas during the previous year.

Asterisks indicate significant differences after the Kruskal-Wallis and the Mann-Whitney-*U* tests (^*^ *p*<0.05, ^**^ *p*<0.01, ^***^ *p*<0.001).

Values marked with the same letter are not significantly different (Dunn’s test, *p*<0.05).

Regarding cultural services, we found two groups of services depending on the respondents’ place of residence. While services such as nature tourism, aesthetic values, environmental education, and the existence value of biodiversity were mostly perceived by urban inhabitants (Mann-Whitney: *U = *919 499.0, *p*<0.0001), recreational hunting and local ecological knowledge obtained higher value scores from inhabitants of rural areas (Mann-Whitney: *U = *1 320 478.0, *p*<0.0001).

People who visited protected areas regularly largely recognized regulating services, while members of environmental or social organizations largely recognized provisioning services (Table 4). Recreational hunting and local ecological knowledge were the most recognized services by rural people (Mann-Whitney: *U = *536 998.0, *p* = 0.012).

Respondents recognized all categories of ecosystem services when they were interviewed in Natural Parks (i.e., medium level of landscape protection) (Table 4). However, provisioning and cultural services were less recognized when respondents were interviewed in National Parks (high level of protection) (Table 4).

### Factors Influencing the Relative Importance People give to Particular Ecosystem Services

**Figure 3:**
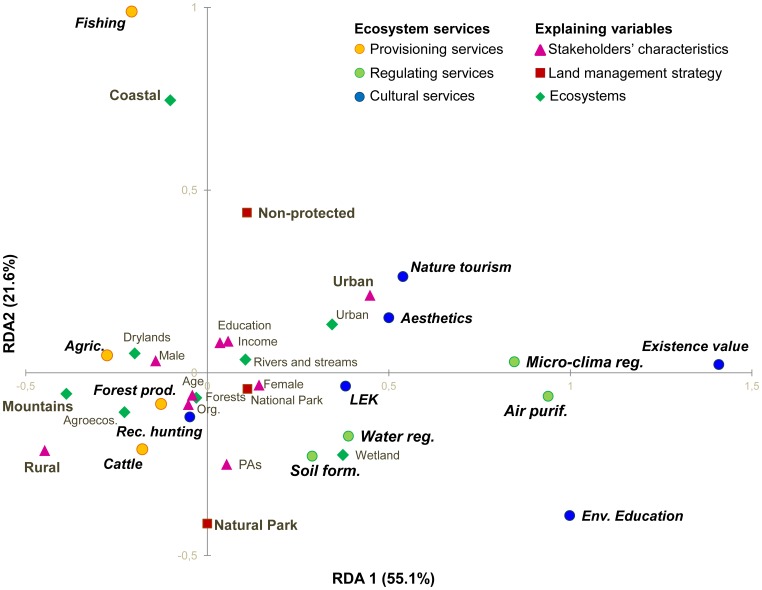
Redundancy analysis biplot. The biplot shows the relationships between stakeholders’ perceptions towards particular ecosystem services and variables related to stakeholders’ characteristics and land management strategies. Grey variables in bold represent explaining variables with higher standardized canonical coefficients for Axes 1 and 2. Detail legend: circles  =  ecosystem services; squares  =  land management strategy (i.e., National Park, Natural Park, or non-protected land); triangles  =  environmental behavior and socio-economic characteristics of stakeholders; diamonds  =  ecosystems. (PAs  =  If respondent visited protected areas during the previous year, LEK  =  local ecological knowledge and sense of place services).

The RDA indicates a statistically significant association between the relative importance of ecosystem services perceived by people and stakeholders’ characteristics, the protection level of ecosystems, and ecosystem type (*p*<0.0001, from 1000 permutations). The first three axes explained 87.8% of the total variance (Table 5). The biplot of the RDA, representing the first two axes, is shown in Figure 3.

**Table 5:** Results of the redundancy analysis.

	Axes 1	Axes 2	Axes 3	Axes 4
Eigenvalue	0.167	0.065	0.034	0.014
Percentage variance explained	55.125	21.572	11.077	4.561
Cumulative % variance explained	55.125	76.697	87.775	92.335
Total inertia	6.760	2.645	1.358	0.559

The first axis of the RDA (55.1% of the variance) revealed a trade-off between provisioning (and recreational hunting) and all other ecosystem services (i.e., regulating and cultural services). RDA1 also revealed different stakeholder perceptions regarding ecosystem services, mainly explained by a rural-urban dichotomy (Figure 3). Rural people most often mentioned provisioning services and recreational hunting. However, urban people reported regulating services that contribute directly to their quality of life in an urban context (i.e., air purification and micro-climate regulation) as well as highly demanded cultural services, including nature tourism, aesthetic values, environmental education and the moral satisfaction obtained from conserving biodiversity (i.e., existence value).

The second axis of the RDA (21.6% of the variance) revealed a gradient of ecosystem services perception related to the level of ecosystem protection, with non-protected areas having positive scores and ‘Natural Parks’ having negative scores. Positive scores were associated with services provided by ecosystems in non-protected areas (e.g., food from agriculture and fishing). Negative scores were associated with services provided by ecosystems in Natural Parks (e.g., forest products, food from cattle, soil formation, water regulation, recreational hunting, and environmental education) (Figure 3).

In addition, the RDA highlights that the respondents’ acknowledgement of particular ecosystem services was associated with specific ecosystem types (Figure 3). For example, food obtained from fishing and shellfishing was strongly related with coastal systems.

### Bundling Ecosystem Services through Social Preferences

**Figure 4:**
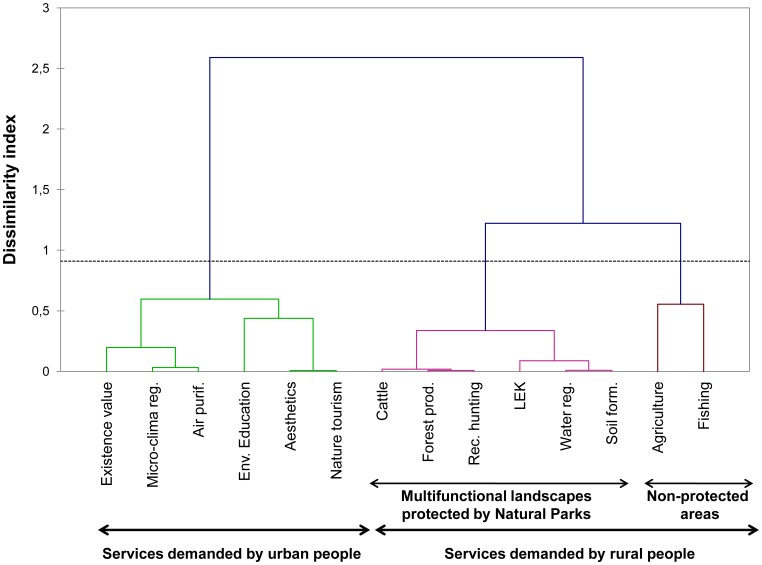
Dendogram of the hierarchical cluster analysis. The dendogram shows ecosystem service bundles resulting from diverging social preferences. Ecosystem service bundles are shown in different colors to improve visualization.

Using the first three axes of the RDA in the hierarchical cluster analysis, we identified three well-defined bundles of ecosystem services (Figure 4). Group I contains ecosystem services demanded by urban populations, including most cultural services, air purification, and micro-climate regulation. Group II represents ecosystem services demanded by rural people in multi-functional landscapes, often protected under the category of Natural Parks. This group contains a high diversity of services, including provisioning (cattle and forest products), regulating (soil formation and water regulation), and cultural services (recreational hunting and local ecological knowledge). Group III contains provisioning services related to food (agriculture and fishing), perceived mostly by rural people and provided mostly by non-protected areas.

## Discussion

### Social Preferences towards Ecosystem Services: A Rural-urban Gradient

Previous studies have found that human preferences toward ecosystem services focus first on provisioning services, followed by regulating services, and finally, on cultural services (e.g., [30]–[32]). In contrast, our results show that regulating services are reported more often than provisioning services, even though the latter are easier to physically identify. Among regulating services, air purification was the service perceived to be the most important, being recognized by 45% of respondents (Table 3). The saliency in the recognition of air quality is consistent with previous studies [8], [31], [33], [34] and may be explained by a combination of factors, including communication media or advertising, education programs [31], and an awareness of high levels of air pollution in Spanish cities [35].

However, social preferences over specific ecosystem services may vary among respondents due to a complex set of factors, including individual needs, cultural traditions, access to ecosystem services, and sources of household income [31]. In fact, these factors are likely to explain a large extent of the contrasting representations of ecosystem services between rural and urban inhabitants. Specific ecosystem services, such as air purification, microclimate regulation, aesthetic value, tourism activities, environmental education, and the existence value of biodiversity were highly valued by urban people (Figure 4) [36]–[38]. However, ecosystem services essential for life, such as food, are less perceived by urban people, despite their increasing dependence on these essential provisioning services [39], [40]. This might happen because dominating worldviews in urban societies have cognitively disconnected their human well-being from life-supporting environments, perceiving ecosystems as external factors to urban people [41], places for enjoying silence [42], aesthetics, and recreational activities [36]. However, the Spanish urban population, which accounts for more than 80% of the total population [43], depends largely on the rural and natural landscapes of Spain, in addition to non-Spanish ecosystems, to satisfy most of its ecosystem service demands [24].

The fact that rural people recognized a highly diversified flow of services (Figure 4) may be because their own well-being is closely connected with more ecosystem services. In fact, particularly in the Mediterranean basin, rural people have acted for centuries as landscape ‘sculptors’, designing multifunctional landscapes that guarantee a diverse flow of ecosystem services [44], [45].

### Synergies among Socio-economic Variables Supporting the Rural-urban Gradient

The rural-urban gradient identified in this study in relation to the social perception of ecosystem services is a consequence of different lifestyles and socio-economic characteristics. Variables such as age, formal education level, gender, and income clearly vary along the rural-urban gradient, having an impact on the rural-urban pattern of ecosystem service perceptions [38], [46] (see Figure 4). For instance, rural areas of Europe suffer a depopulation process as younger people migrate to cities, resulting in aging of the rural population [46]. Our results show that elderly people from rural areas were more aware of provisioning services and recreational hunting because their lifestyle is more likely to depend on the primary sector and their understanding of ecosystem services is based on experiential local knowledge related to traditional agroecological activities [26], [47]. In contrast, the perception of ecosystem services by younger urban people is mediated more by formal education [48] (Figure 3). In fact, higher education levels of younger people are associated with a higher perception of environmental education as an important ecosystem service. This synergy reinforces people’s attitudes in valuing services such as nature tourism, aesthetics, and the existence value of biodiversity (Figure 4). Similarly, higher education levels combined with environmental behavior increases the probability that people acknowledge an ecosystem’s capacity to provide services (Table 2).

Additionally, we found that gender roles are significant in defining preferences toward ecosystem services. In contrast to previous studies on the topic, which have shown that females perceived fewer ecosystem services than males [31], [49], [50], our results show that females have a higher probability of perceiving an ecosystem’s capacity to provide services (Table 2). Moreover, while men were more likely to perceive provisioning services, women were more likely to perceive regulating services (Table 4). These findings are in accordance with previous research on gender and pro-environmental behaviors, which have found that women exhibit more environmental behavior than men (e.g., [51]–[54]). Gender differences on ecosystem service awareness could be explained by the gender-differentiated roles in agroecological labor, expertise, and knowledge [55], [56], as well as by the masculinization phenomenon taking place in Spanish rural areas [43].

### The Importance of Formal vs. Non-formal Education in Shaping People’s Perception of Ecosystem Services

Our results suggest that different types of knowledge may be required to capture the entire range of services that ecosystems provide, i.e., experiential or local knowledge (non-formal) and technical or experimental knowledge (formal) [26], [47]. Previous studies have shown that both types of knowledge are complementary and that their combination might play a positive role in sustaining the delivery of multiple ecosystem services (e.g., [57], [58]). Here, we found that formal knowledge associated with environmental education service is linked to urban worldviews, whereas local ecological knowledge is related to rural worldviews. Both types of knowledge are bundled with different ecosystem services (Figure 4). Environmental education service is bundled with cultural services associated with recreational activities and aesthetic benefits. In contrast, local ecological knowledge is bundled with services related to multifunctional landscapes, i.e., soil formation, water regulation, forest products, recreational hunting, and food from cattle (Figure 4). In fact, in the Mediterranean agro-silvo-pastoral systems, local ecological knowledge has played a crucial role for centuries in developing management practices that secure essential ecosystem service supplies for maintaining their livelihood [45].

The fact that local ecological knowledge is bundled with regulating services related to water and soil suggests that most traditional land management practices in Spain focus on managing these ecosystem components to tackle soil erosion, aridity, drought, and flooding [59]. However, this Mediterranean social-ecological memory is currently endangered along with the ecosystem services delivered by multifunctional landscapes in Spain [24] due to the increasing land homogenization process, as a consequence of global market integration, mechanization, rural abandonment, and strict biodiversity conservation policies [45].

### Land-use Management Matters

It is broadly recognized that over the past century most landscape management strategies have favored the delivery of provisioning services at the expense of regulating and cultural services [60]–[62]. Rodríguez et al. [63] noted that a strong emphasis on provisioning services in land-use planning is likely related to the fact that their value is more tangible and identifiable by society. Interestingly, our results found the opposite trend, with people placing higher value on regulating and cultural services than provisioning services.

To a large extent, the institutional response to accelerate land use change that promotes provisioning services has led to a parallel increase in biodiversity conservation policies through the establishment of protected areas [60], [64]. However, in Spain, land disuse because of strict protected areas and land overuse through land intensification in non-protected areas, have affected the provision of a diverse flow of ecosystem services [24], [45]. The multifunctionality of the Mediterranean agro-silvo-pastoral systems is declining due to homogenization as a result of landscape intensification, rural abandonment, and strict conservation policies, which can decrease biodiversity and ecosystem services [65], [66].

Rather than supporting the revitalization of traditional rural practices, institutional responses to land-use change have favored the demands of an urban conservation movement through the creation of strict protected areas, i.e., National Parks [46]. The lack of incentives to maintain traditional management practices, due to both market integration and strict conservation policies, has resulted in a territorial matrix in which strict conservation takes place inside National Parks while land intensification occurs at their borders, establishing a conservation versus development model [11], [67]. This territorial management model leads to loss of multifunctional landscapes and diversity in the flow of ecosystem services [24]. Indeed, multifunctional landscapes with associated cultural values and traditional practices (in this study, referring to Natural Parks) are perceived by people to supply a more diverse flow of services (Table 4). On the contrary, respondents in urban and industrialized areas perceived mostly cultural services, primarily tourism, aesthetics, environmental education, or existence value; and people interviewed in intensively managed rural areas mostly perceived services related to food (Figure 4). In fact, while regulating and cultural services related to traditional practices are mostly perceived in multi-functional land-use areas, food-related provisioning services are primarily perceived from mono-functional, intensively managed agricultural areas. Tourism and aesthetics play a central role in urban areas [68].

### Concluding Remarks

Recent contributions have stressed the need to advance our understanding of the social values of ecosystem services, trade-offs resulting from different interests in ecosystem use, and ecosystem service bundles promoted by different landscape management strategies [62]. Our results have increased our understanding of the socio-cultural values of ecosystem services by empirically demonstrating the following: (*i*) different stakeholders hold different values and perceptions toward ecosystem services; (*ii*) there is an important rural-urban gradient in preferences toward ecosystem services based on gender, different lifestyles, and different sources of knowledge regarding ecosystem services (i.e., formal education versus local knowledge [26], [47]) (Figure 3); (*iii*) local ecological knowledge is bundled with key regulating services related to ecosystem functioning (i.e., soil formation and water regulation) (Figure 4); (*iv)* a gender-differentiated role exists regarding perceptions of ecosystem services (Table 2 and 4); (*v*) the perception of ecosystem services may vary as a consequence of land management strategy (i.e., National Park, Natural Park, and non-protected areas) (Table 4); (*vi*) trade-offs can be identified from socio-cultural preferences as people’s willingness to trade-off conservation of one ecosystem service against another [26]; and (*vii*) ecosystem service bundles can be identified from people’s systemic representations of interrelationships between ecosystem services (Figure 4).

Although different studies have recognized that ecosystem service assessments should incorporate ecological, socio-cultural, and monetary values [10], [69], most studies restrict their analysis to biophysical and monetary factors [12], [17], leaving the assessment of the socio-cultural values of ecosystem services largely unaddressed. Overlooking social awareness of ecosystem services can blind society to the variety of services provided by ecosystems and can act as an obstacle for mainstreaming ecosystem services across societal sectors. Ecosystem service trade-offs emerge as societies modify landscapes because of their different perceptions, interests and values. As a result, ecosystem service evaluations should incorporate stakeholders’ representations and their intangible values of the ecosystems, i.e., socio-cultural preferences [70]. Visualizing ecosystem service trade-offs based on socio-cultural preferences can serve as a tool to identify the impact of different management options on an ecosystem’s capacity to deliver services and as a basis for decision-making processes. Therefore, ecosystem service assessments should incorporate non-monetary methods to assess social preferences in order to identify relevant services for people [17], potential social conflicts due to different needs and perceptions, trade-offs among ecosystem services, and ecosystem service bundles.

## Supporting Information

Figure S1 **Characteristics of study sites in relation to climate data in Spain.**
(PDF)

Table S1 **Main characteristics of sites included in this study.**
(PDF)

Table S2 **Summary statistics of socio-economic characteristics of polled people for each case site.**
(PDF)

Table S3 **Summary of the variables obtained from the questionnaire and used for the different analysis performed in the study.**
(PDF)
