# Supplementary material for: Uncovering Ecosystem Service Bundles through Social Preferences
Source: PLoS One. 2012 Jun 18;7(6):e38970. doi: 10.1371/journal.pone.0038970 (PMC3377692; doi:10.1371/journal.pone.0038970)
Supplement: Figure S1 — Characteristics of study sites in relation to climate data in Spain. (PDF) [file pone.0038970.s001.pdf]

Figure S1.

Characteristics of study sites in relation to climate data in Spain.

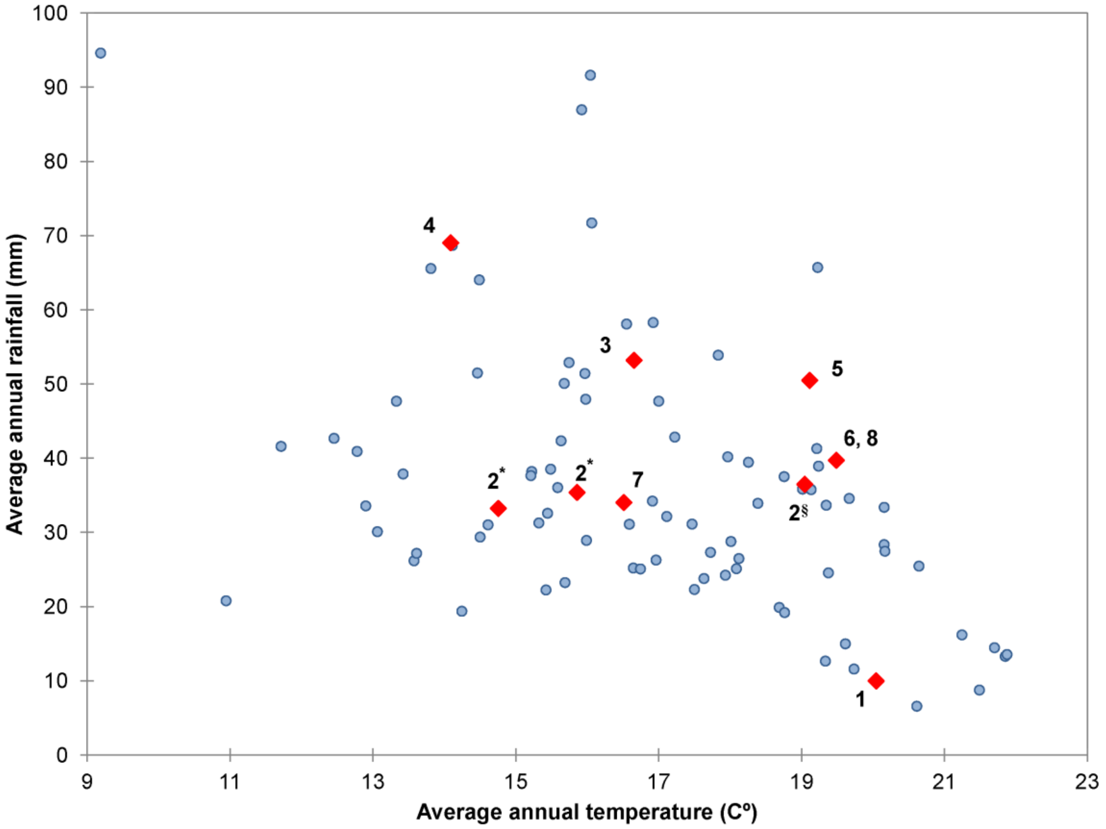

Blue circles are data of different climate stations in Spain.

Red diamonds are climatic data of study sites. Study sites are: 1 = The Adra River watershed, 2* = The Conquense Drove Road (wintering areas), 2§ = The Conquense Drove Road (summering areas), 3 = The Bilbao Metropolitan Greenbelt, 4 = Costa da Morte, 5 = Doñana, 6 = The Guadiamar Green Corridor, 7 = Sierra Nevada Mountains, and 8 = Sierra Norte de Sevilla.
