# Supplementary material for: Uncovering Ecosystem Service Bundles through Social Preferences
Source: PLoS One. 2012 Jun 18;7(6):e38970. doi: 10.1371/journal.pone.0038970 (PMC3377692; doi:10.1371/journal.pone.0038970)
Supplement: Table S1 — Main characteristics of sites included in this study. (PDF) [file pone.0038970.s002.pdf]

Table S1. Main characteristics of sites included in this study and data sampling characteristics in each case study.

Site	Characteristics of the case study						Data sampling characteristics		
	Total area (km^2)	*Altitude range (m.a.s.l.)*	*Main ecosystems[*]*	*Type of Protected Area*	*Nº of munic.*	*Population density (inhabitants km^{-2})*	*Nº of sampling points*	*Date of sampling*	*Sample size (N)*
1. The Adra River watershed	744	0 –2611	Drylands<br>Forests<br>Mountains<br>Rivers and streams	The upper area was declared National Park in 1999 and the Albuferas of Adra were declared Natural Park in 1989	14	39	20	May 2009 - March 2010	200
2. The Conquense Drove Road	15 297	270 - 1930	Agroecosystems<br>Forests	There are 5 Natural Parks along its extension	77	26	39	September 2009 - March 2010	416
3. The Bilbao Metropolitan Greenbelt	413	0 – 998	Urban<br>Forests	-	29	2164	23	May 2009 - July 2010	500
4. Costa da Morte	1600	0 –580	Coastal<br>Agroecosystems	-	17	80	7	September 2008	212
5. Doñana	3298	0 –280	Wetlands<br>Coastal<br>Rivers and streams<br>Forests	It was declared National Park in 1969 and their surroundings were declared Natural Park in 1980	16	65	17	October 2007 -March 2009	772
6. The Guadiamar Green Corridor	2059	0 –580	Rivers and streams<br>Agroecosystems	The lower area was declared National Park in 1969 and Natural Park in 1980	15	44	10	October 2008 - March 2009	215
7. Sierra Nevada mountains	3655	180 –3479	Mountains<br>Forests<br>Drylands	It was declared National Park in 1999 and in 1989 its surroundings were designated as a Natural Park	73	94	59	May 2009 - July 2011	657
8. Sierra Norte de Sevilla	1775	35 - 945	Agroecosystems<br>Forests	It was declared Natural Park in 1989	10	12	6	July 2007	407

[*] Based on Millennium Ecosystem Assessment classification [1]

References

1. Millennium Ecosystem Assessment (MA) (2005) Ecosystems and Human Well-being: Synthesis. Washington, DC.: Island Press. 137 p.
