# Supplementary material for: Uncovering Ecosystem Service Bundles through Social Preferences
Source: PLoS One. 2012 Jun 18;7(6):e38970. doi: 10.1371/journal.pone.0038970 (PMC3377692; doi:10.1371/journal.pone.0038970)
Supplement: Table S2 — Summary statistics of socio-economic characteristics of polled people for each case site. (PDF) [file pone.0038970.s003.pdf]

Table S2. Summary statistics of socio-economic characteristics of polled people for each case site.

	N	Level of studies (%)				Age (years)	Income (€)	Gender (%)		People living in a rural or urban municipality (%)		Member of an association[*] (%)	Visiting PAs[†] during the last year (%)
		Non	*Primary*	*Secondary*	*University*			*Male*	*Female*	*Rural*	*Urban*		
1. The Adra River watershed	200	2.0	20.5	24.5	53.0	40.3	1416.0	59.0	41.0	41.5	58.5	16.5	62.0
2. The Conquense Drove Road	416	3.6	29.1	32.5	34.9	41.7	1235.6	58.9	41.1	63.0	37.0	44.5	72.4
3. The Bilbao Metropolitan Greenbelt	500	0.6	22.8	36.6	40.0	41.9	1212.9	58.2	41.8	8.8	91.2	7.2	60.8
4. Costa da Morte	212	8.0	26.4	42.9	22.6	36.7	1357.9	64.6	35.4	87.7	12.3	3.3	-
5. Doñana	772	10.0	25.1	24.6	40.3	41.8	1557.2	58.4	41.5	30.8	69.2	16.5	56.4
6. The Guadiamar Green Corridor	215	8.8	37.2	32.6	21.4	42.3	1638.7	65.1	34.9	64.7	35.3	7.9	-
7. Sierra Nevada mountains	657	2.1	7.0	17.5	73.4	40.1	1278.4	63.5	36.5	97.6	2.4	8.8	81.4
8. Sierra Norte de Sevilla	407	6.1	22.6	48.2	22.4	37.7	1114.5	51.8	48.2	74.9	25.1	3.7	38.1
All sample	*3379*	*5.1*	*22.0*	*30.5*	*42.3*	*40.6*	*1310.4*	*59.5*	*40.5*	*56.2*	*43.8*	*13.9*	*54.7*

[*] Including environmental or social association
[†] PAs = protected areas
