# Supplementary material for: Uncovering Ecosystem Service Bundles through Social Preferences
Source: PLoS One. 2012 Jun 18;7(6):e38970. doi: 10.1371/journal.pone.0038970 (PMC3377692; doi:10.1371/journal.pone.0038970)
Supplement: Table S3 — Summary of the variables obtained from the questionnaire and used for the different analysis performed in the study. (PDF) [file pone.0038970.s004.pdf]

Table S3. Summary of the variables obtained from the questionnaire and used for the different analysis performed in the study.

Code of variable	Description	Type	Attributes	Analyses
**Dependent variables**				
Service	If respondent recognized ecosystems' capacity to provide services	Dummy	1: yes; 0: no	Logit<br>Chi-squared
Serv_cat	Relative importance held to each of the services categories	Continuous		Kruskal-Wallis<br>Mann-Whitney
Category of ecosystem services identified				
Prov<br>*Reg*<br>*Cult*	Category of ecosystem service perceived as important by respondent	Nominal	Provisioning<br>Regulating<br>Cultural	Chi-squared
Particular ecosystem service identified as important				
	If respondent recognized ecosystems' capacity to provide...			
Agric	food from agriculture	Dummy	1: yes; 0: no	RDA*
Cattle	food from cattle	Dummy	1: yes; 0: no	RDA
Fishing	food through fishing and shellfishing activities	Dummy	1: yes; 0: no	RDA
Forest prod.	forest resources	Dummy	1: yes; 0: no	RDA
Micro-clima	micro-climate regulation	Dummy	1: yes; 0: no	RDA
Air purif.	air purification	Dummy	1: yes; 0: no	RDA
Water reg.	hydrological regulation and water depuration	Dummy	1: yes; 0: no	RDA
Soil form.	soil fertility and erosion control	Dummy	1: yes; 0: no	RDA
Tourism	nature tourism and ecotourism	Dummy	1: yes; 0: no	RDA
Aesthetic	aesthetic values from landscape enjoy	Dummy	1: yes; 0: no	RDA
Env. Educ.	possibility of environmental education	Dummy	1: yes; 0: no	RDA
LEK	local ecological knowledge and sense of place	Dummy	1: yes; 0: no	RDA
Rec. hunting	recreational hunting	Dummy	1: yes; 0: no	RDA
Existence value	Moral satisfaction obtained from protecting biodiversity†	Dummy	1: yes; 0: no	RDA
**Independent variables**				
Management strategy				
	If the sampling point is...			
National Park	inside borders of National Park (representing the highest level of protection)	Nominal		Kruskal-Wallis
Natural Park	inside borders of Natural Park (with medium level of protection)			RDA
Non-protected	outside PA‡ (without protection)			

* RDA = Redundancy analysis
† Adapted from [1]
‡ PA = Protected Area

Environmental behaviour				
PAs	If respondent visited PAs during the previous year	Dummy	1: yes; 0: no	Logit<br>Mann-Whitney<br>RDA
Organization	If respondent held membership of environmental or social organization	Dummy	1: yes; 0: no	Logit<br>Mann-Whitney<br>RDA
Socio-demographic characteristics				
Place of residence	If respondents lived in a rural or urban municipality according to the definition of the Spanish Law for the Sustainable Development of the Rural Environment (Law 45/2007)	Nominal	Rural<br>Urban	Mann-Whitney<br>RDA
Education	Respondents' formal studies level	Ordinal	0: none; 1: primary; 2:secondary; 3: university	Logit<br>Kruskal-Wallis
		Continuous	Ln (study level)	RDA
Age	Younger people (age < 30)	Dummy	1: yes; 0: no	Mann-Whitney
	Older people (age > 70)	Dummy	1: yes; 0: no	Mann-Whitney
	Respondent's age	Continuous	Ln (Age)	RDA
Gender		Nominal	Male<br>Female	Logit<br>Mann-Whitney<br>RDA
Income	Monthly income of respondent	Continuous	Ln (monthly income)	RDA
Ecosystems§	If the sampling point is in or close to...			
Rivers and streams	Rivers and streams	Dummy	1: yes; 0: no	Chi-squared<br>RDA
Wetlands	Wetlands	Dummy	1: yes; 0: no	Chi-squared<br>RDA
Coastal	Coastal systems	Dummy	1: yes; 0: no	Chi-squared<br>RDA
Mountains	Mountains (altitude ≥ 2000 m.a.s.l.)	Dummy	1: yes; 0: no	Chi-squared<br>RDA
Forests	Forests	Dummy	1: yes; 0: no	Chi-squared<br>RDA
Drylands	Drylands	Dummy	1: yes; 0: no	Chi-squared<br>RDA
Agroecosystems	Agroecosystems	Dummy	1: yes; 0: no	Chi-squared<br>RDA
Urban	Urban systems	Dummy	1: yes; 0: no	Chi-squared<br>RDA

§ Based on Millennium Ecosystem Assessment classification [2]

References

1. Kahneman D, Knetsch JL (1992) Valuing public goods: The purchase of moral satisfaction. Journal of Environmental Economics and Management 22: 57–70.

2. Millennium Ecosystem Assessment (MA) (2005) Ecosystems and Human Well-being: Synthesis. Washington, DC.: Island Press. 137 p.
